# Integration of HiMoRNA and RNAChrom: Validation of the Functional Role of Long Non-coding RNAs in the Epigenetic Regulation of Human Genes Using RNA-Chromatin Interactome Data

**DOI:** 10.32607/actanaturae.27543

**Published:** 2025

**Authors:** I. S. Ilnitskiy, G. K. Ryabykh, D. A. Marakulina, A. A. Mironov, Yu. A. Medvedeva

**Affiliations:** Lomonosov Moscow State University, Faculty of Bioengineering and Bioinformatics, Moscow, 119234 Russia; Vavilov Institute of General Genetics, Russian Academy of Sciences, Moscow, 119991 Russia; Skryabin Institute of Bioengineering, FRC Biotechnology, Russian Academy of Sciences, Moscow, 117312 Russia; School of Biomedical Physics, Moscow Institute of Physics and Technology, Dolgoprudny, Moscow, 141701 Russia

**Keywords:** long non-coding RNA, histone modification, RNA-chromatin interaction

## Abstract

Long non-coding RNAs (lncRNAs) play a crucial role in the epigenetic regulation of gene expression by recruiting chromatin-modifying proteins to specific genomic loci. Two databases, previously developed by our groups, HiMoRNA and RNA-Chrom, provide valuable insights into this process. The former contains data on epigenetic modification regions (peaks) correlated with lncRNA expression, while the latter offers genome-wide RNA–chromatin interaction data for tens of thousands of RNAs. This study integrated the two resources to generate experimentally supported, interpretable hypotheses regarding lncRNA-mediated epigenetic gene expression regulation. We adapted the web interfaces of HiMoRNA and RNA-Chrom to enable the retrieval of chromatin contacts for each “lncRNA–pigenetic modification–ssociated gene” triad from HiMoRNA, either at specific genomic loci or genome-wide via RNA-Chrom. The integration analysis revealed that for the lncRNAs MALAT1, HOXC-AS2, NEAT1, NR2F1-AS1, PVT1, and MEG3, most HiMoRNA peaks are located within 25 kb of their RNA-Chrom contacts. Further investigation confirmed the RNA–hromatin contacts of MIR31HG and PVT1 lncRNAs, with HiMoRNA peaks for H3K27ac and H3K27me3 marks in the loci of the genes *GLI2 *and *LATS2*, respectively, which are known to be regulated by these RNAs. Thus, the integration of HiMoRNA and RNA-Chrom offers a powerful platform to elucidate the role of specific lncRNAs in the regulation of histone modifications at both individual loci and genome-wide levels. We expect this integration to help significantly advance the functional annotation of human lncRNAs.

## INTRODUCTION

Human cells transcribe a vast number of long non-coding RNAs (lncRNAs), with their quantity comparable to that of protein-coding genes [1, 2]. Functional annotation of lncRNAs is challenging due to their low expression levels, tissue specificity, and low sequence conservation [3, 4, 5]. Nevertheless, lncRNAs were observed to preserve certain characteristics, notably synteny with neighboring genes, secondary structure, and similarity in short sequence fragments [6]. In addition, the transcriptional regulation of lncRNA transcription exhibits intricacy comparable to that of protein-coding RNAs, facilitating their involvement in diverse molecular processes [7]. Suppression of lncRNAs has been shown to result in significant changes in the transcriptional profile of cells [8]. These findings indicate a functional role for numerous lncRNAs. Most lncRNAs were shown to interact with chromatin and to be involved in the epigenetic regulation of genomic loci and the structural organization of chromosomes [9, 10, 11, 12]. Accordingly, identifying the functional genomic targets of chromatin- associated lncRNAs is of high value.

Previously, we developed the HiMoRNA database [13], which catalogues over 5 million epigenetic “peaks,” genomic regions exhibiting one of 10 histone modifications, with their modification level significantly correlating with lncRNA expression across at least 20 cell lines and tissues. Where possible, histone modification peaks in HiMoRNA are associated with genes, forming a triad: “lncRNA–epigenetic modification peak–associated gene.” Within such a triad, the lncRNA is hypothesized to modulate the expression of the corresponding gene through the promotion or repression of histone modification within the gene’s associated peak region. In the case of promotion, peaks positively correlate with lncRNA expression (“+” peaks), while in the case of repression, the correlation is negative (“–” peaks). These associations enable the formulation of hypotheses regarding the role of lncRNAs in the modulation of epigenetic modifications at specific genomic loci and the regulation of gene expression. However, to formulate and empirically test reasonable hypotheses, the 5-million- peak dataset has to be pre-processed to select the most reliable peaks. Experimental methods for detecting RNA-chromatin interactions can provide valuable data for this purpose. Several experimental methods exist to identify chromatin regions interacting with non-coding RNAs. These methods can be broadly classified into two categories: “one-to-all” [11, 14, 15, 16, 17, 18], which identify the contacts of a specific RNA with chromatin, and “all-to-all” [19, 20, 21, 22, 23, 24], which capture all possible RNA–DNA contacts in a cell [25]. Notwithstanding their utility, these approaches are prone to high false positive rates. Additionally, “all-toall” methods show low sensitivity to lowly expressed RNAs and bias toward nascent transcripts. Despite these challenges, genome-wide data on non-coding RNA (ncRNA) interactions with chromatin are crucial for elucidating their mechanisms of action. In this context, the RNA-Chrom database [26] was recently created. It contains experimental data on thousands of RNA-chromatin contacts and offers two analytical modes (“from RNA” and “from DNA”) that can be used for research purposes.

To improve and streamline the functional annotation of lncRNAs, we have integrated the HiMoRNA and RNA-Chrom databases. The web interfaces of HiMoRNA and RNA-Chrom were modified to provide direct access to chromatin contacts for 4,124 out of the 4,145 lncRNAs from HiMoRNA in RNA-Chrom. This integration enables the generation of hypotheses regarding the mechanisms of epigenetic regulation of human gene expression by long non-coding RNAs, supported by experimental data on their interactions with chromatin. We anticipate that this unified resource will prove a valuable tool for identifying high-confidence “lncRNA–pigenetic modification– associated gene” triads for further experimental investigation of lncRNA mechanisms in gene regulation. The HiMoRNA database is available to users at https://himorna.fbras.ru (as of 20.10.2024).

## EXPERIMENTAL PART

**Integration of the HiMoRNA and RNA-Chrom databases**

**Fig. 1:**
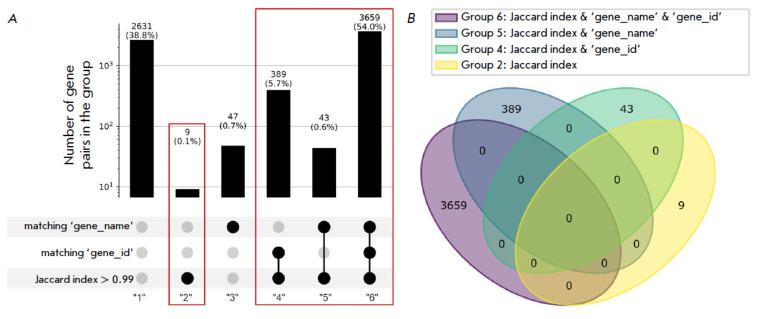
Intersection of 4,145 genes from HiMoRNA with 60,619 genes from RNA-Chrom. (A) Division of gene pairs into six groups based on the similarity metrics they satisfy. Groups achieving unambiguous gene correspondence are highlighted with red rectangles. (B) Venn diagram showing the overlap between the gene groups 2, 4, 5, and 6 (the total number of gene pairs in these four groups is 4,100)

**Fig. 2:**
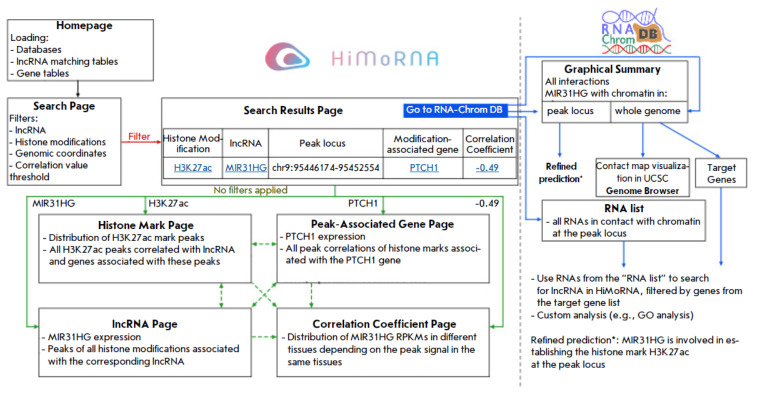
Usage scenario of the HiMoRNA and RNA-Chrom databases after integration. Rectangles represent web pages, and arrows indicate movement between them

Due to differences in gene annotation sources (HiMoRNA uses GENCODE v31, while RNA-Chrom uses GENCODE v35), we established gene correspondence using three similarity metrics: (1) matching gene names (‘gene_name’, *Fig. 1A*); (2) matching gene identifiers (‘gene_id’, *Fig. 1A*); and (3) a Jaccard index (the ratio of the length of gene overlap to the length of their union) greater than 0.99 (Jaccard index > 0.99, *Fig. 1A*). Due to differences in naming conventions and genomic coordinates between annotation versions, gene identifiers and positions often do not align directly. To address this issue, we intersected 4,145 lncRNA genes from HiMoRNA with 60,619 genes from RNA-Chrom based on genomic coordinates using the “*intersect*” command from “*bedtools”*. This yielded 6,778 gene pairs, exceeding the number of HiMoRNA entries, because some HiMoRNA genes intersected multiple times with RNA-Chrom genes. The two HiMoRNA genes (ENSG00000267034.1 and ENSG00000280076.1) did not intersect with any RNA-Chrom genes. Subsequently, the Jaccard index was calculated for each gene pair. The gene pairs were classified into six groups based on similarity metrics (*Fig. 1A*). By using a Jaccard index > 0.99 as the primary similarity metric, we identified four groups (groups 2, 4, 5, and 6) as having no overlapping genes (*Fig. 1A*), establishing 4,100 unambiguous gene correspondences. For the remaining 43 genes from HiMoRNA, 24 additional matches to RNA-Chrom genes were established using the ‘gene_name’ matching metric. In total, we identified 4,124 lncRNA genes common to both databases (see *Supplementary Table 1* for a full correspondence table that is available for download on the HiMoRNA web resource).

To support database integration and streamline access to chromatin interaction data, we made several enhancements to the RNA-Chrom and HiMoRNA interfaces. These include parameter processing (locus, RNA name, RNA-Chrom internal RNA identifier, organism) from a specific type of URL (e.g., https://rnachrom2.bioinf.fbb.msu.ru/basic_graphical_summary_dna_filter?locus=chrX:23456-24253566&name=XIST&rnaID=227896&organism=Homo+sapiens) and providing information on the chromatin contacts of the requested lncRNA across different experiment types on a new browser page.

Enhancements to the HiMoRNA interface include the incorporation of the gene correspondence table between RNA-Chrom and HiMoRNA to ensure correct URL generation.

A “Go to RNA-Chrom DB” button with a dropdown menu (*Fig. 2*) was added to the “Search Results Page,” allowing the user to generate three types of URL links to navigate to the RNA-Chrom page:

a) Contacts of the given lncRNA in a specific genomic locus, extended by 1 / 5 / 10 / 25 / 50 / 100 kb;

b) All contacts of the given lncRNA;

c) All RNAs with contacts in a specific genomic locus.

**One-sided Fisher’s exact test**

In most triads, histone modification peaks show both negative and positive correlations between lncRNA expression and the peak signal level (hereinafter denoted “–” and “+” peaks, respectively). The presence of a “+” peak suggests a role for the lncRNA in establishing the histone modification, whereas a “–” peak implies its role in removing the modification.

The alignment of predictions with published experimental results was evaluated by selecting lncRNAs and their corresponding histone peaks (positively and negatively correlated), extended by ±25 kb, and filtering for those where the proportion of peaks supported by contacts for at least one histone mark exceeded 40%. We subsequently conducted independent righttailed and left-tailed Fisher’s exact tests for each lncRNA-histone mark pair. For example, a representative contingency table for the “PVT1–H3K27ac” pair is provided in *Supplementary Table 2.*

**Red-ChIP data**

As a case study, we used the lncRNA PVT1 to validate integration between HiMoRNA and RNA-Chrom using independent experimental data. Specifically, we incorporated Red-ChIP data [27], available in the Gene Expression Omnibus under accession number GSE174474, samples GSM5315228 and GSM5315229 (hES cell line). The Red-ChIP method captures RNAchromatin contacts mediated by the EZH2 protein, a component of the PRC2 complex, which establishes, among others, the H3K27me3 histone modification.

Primary data processing followed the established RNA-Chrom database protocol. Primary data processing followed the established RNA-Chrom database protocol. Subsequently, we identified genomic regions enriched with lncRNA PVT1 chromatin contacts using the BaRDIC program (--qval_type all; --qval_threshold 1) [28]. Consequently, 3,242 genomic regions exhibiting potential functionality and EZH2-mediated PVT1 binding were identified.

## RESULTS

**Integration of Databases**

Given that HiMoRNA contains millions of epigenetic peaks, selecting the most reliable ones for further analysis is critical. For this task, we integrated HiMoRNA peak data with RNA-chromatin interactome data from the RNA-Chrom database. This integration was achieved by establishing one-to-one correspondences between genes in both databases and modifying their web interfaces (see “Experimental Section,” subsection “Integration of HiMoRNA and RNA-Chrom Databases”). This strategy allows HiMoRNA to generate specific URL queries for 4,124 out of the 4,145 lncRNAs listed in RNA-Chrom, enabling, in particular, the identification of other chromatin loci with which the investigated RNA interacts. This approach significantly expands our understanding of the function of a specific RNA.

The general integration scheme is presented in *Fig. 2*. To utilize the integration, the user first needs to find the target lncRNA in the HiMoRNA database. From the HiMoRNA homepage, users can download the database itself, as well as the “Gene Table” and the “lncRNA Correspondence Table” added as part of the integration, to search for genes/lncRNAs of interest by genomic coordinates. This feature accommodates potential mismatches between user-specified Ensembl identifiers/lncRNA names and those present in the HiMoRNA database. On the search page, the user must configure filters according to their task, specifying the lncRNAs, histone modifications, genomic coordinates, and genes associated with the selected histone modifications.

On the search results page, users may conduct a more thorough examination of the retrieved predictions, including accessing the RNA-Chrom database. To do this, the user should select the desired “lncRNA–epigenetic modification peak–associated gene” triad from the interactive results table and then click the “Go to RNA-Chrom DB” button. In the dropdown menu, the user should select the appropriate option to navigate to a page displaying: 1) contacts of the given lncRNA in the region of a specific peak (with the option to choose how much to extend the peak coordinates when searching for contacts); 2) all contacts of the given lncRNA; or 3) all lncRNAs with contacts in the specified locus. The user will be automatically redirected to a graphic summary of the lncRNA-chromatin interactome on the RNA-Chrom database webpage. This summary allows the user to determine whether the functional relationship “lncRNA–pigenetic modification” from HiMoRNA is mediated by the physical presence of the lncRNA at the corresponding genomic locus, as well as to identify other lncRNAs potentially involved in the regulation of that locus. Visual analysis is enabled by utilizing the UCSC Genome Browser to load data from all relevant experiments by clicking “VIEW IN GENOME BROWSER.” By selecting a single RNA-chromatin interactome experiment, the user can obtain a list of the genes located in the genomic region of interest, along with statistics on their interactions with the lncRNA, by clicking “ALL TARGET GENES.” This list of genes can be downloaded for further research, such as performing a GO analysis. The “Use Cases” section offers a comprehensive discussion and exemplification of the HiMoRNA and RNA-Chrom database integration.

**Consistency of HiMoRNA and RNA-Chrom Results**

**Fig. 3:**
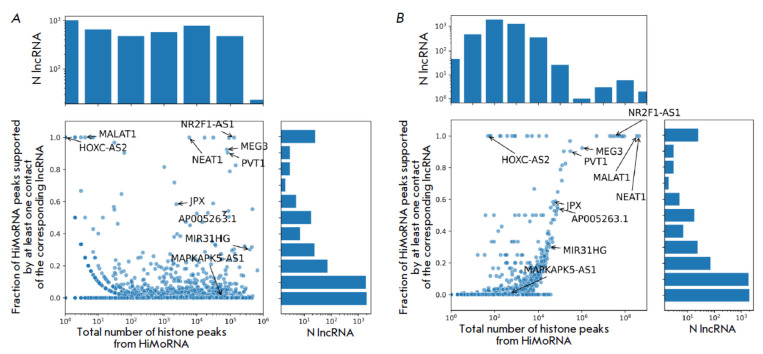
Proportions of HiMoRNA peaks confirmed by at least one contact of the corresponding lncRNA from RNA-Chrom, relative to the total number of HiMoRNA peaks for the corresponding lncRNA (A) and the total number of contacts for the corresponding lncRNA from RNA-Chrom (B). Genomic coordinates of contacts are extended by ±25 kb

To assess the completeness of the integration, we analyzed the frequency of confirmation of histone peaks from HiMoRNA, correlated with lncRNA expression, using data on the corresponding lncRNA chromatin contacts from RNA-Chrom. Out of the 4,145 lncRNAs present in HiMoRNA, 4,011 (96.8%) were found to have at least one contact in the RNA-Chrom database, with 29 RNAs not matching between the databases and 105 (2.5%) having no contacts in RNA-Chrom. Among the 4,011 lncRNAs of interest, only 35.5% had at least one peak supported by the contacts of the corresponding lncRNA. However, due to the design of experimental protocols, actual lncRNA-chromatin interactions may occur at a distance from the experimentally detected contact. To address this issue, we extended the contact coordinates for a more accurate assessment of the correspondence between HiMoRNA predicted peaks and RNA-Chrom data. Extending contact ranges by ±1, ±5, ±10, ±25, and ±50 kb resulted in a respective increase in the percentage of RNAs with HiMoRNA peaks confirmed by at least one contact to 38.5%, 42.7%, 45.7%, 50.1%, and 53%. Some lncRNAs (e.g., MALAT1, HOXC-AS2, NEAT1, NR2F1-AS1, PVT1, MEG3) had nearly all HiMoRNA peaks confirmed with ±25 kb extension (*Fig. 3*). However, lncRNAs with a significantly lower proportion of peaks extended by ±25 kb and confirmed by contacts are more common (e.g., JPX, AP005263.1, MIR31HG) or have a proportion approaching 0 (e.g., MAPKAPK5-AS1). This discrepancy likely arises from the incomplete datasets of lncRNAs in HiMoRNA and RNA-Chrom, resulting from the stringent criteria used for prediction filtering and the limitations inherent in available experimental RNA-chromatin interaction data. For example, in RNA-Chrom, half of the lncRNAs considered in this study have fewer than 200 contacts (*Fig. 3B*), as most lncRNAs rely on “ll-to-all”experimental data, which insufficiently captures contacts of lowly expressed RNAs.

**Fig. 4:**
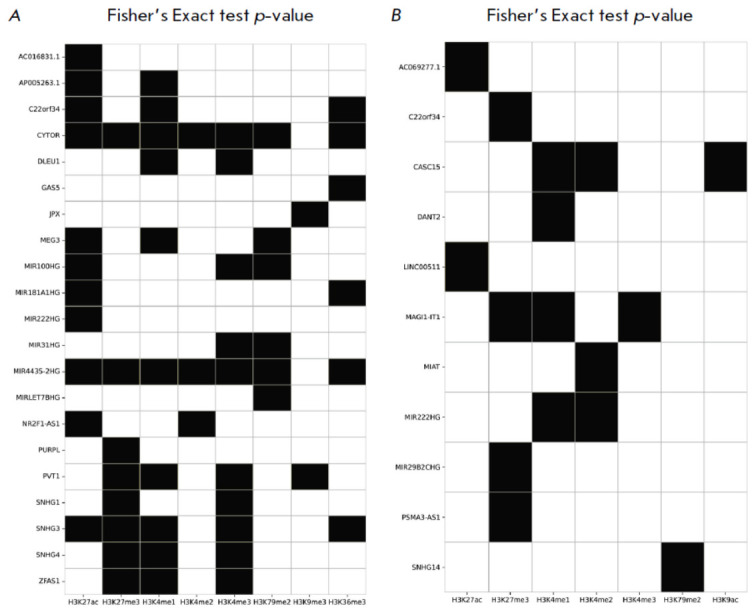
Heatmap showing the results of Fisher’s exact test for pairs of “lncRNA – histone mark peaks extended by ±25 kb”. Black indicates that the proportion of “–” or “+” histone peaks supported by contacts of the corresponding lncRNA is greater than 0.4 and the p-value of the Fisher’s exact test is less than 10-3; otherwise, it is white. (A) Right-tailed Fisher’s test: “+” peaks of corresponding histone marks are better supported by RNA-Chrom contacts than “–” peaks. (B) Left-tailed Fisher’s test: “–” peaks of corresponding histone marks are better supported by RNA-Chrom contacts than “+” peaks

HiMoRNA triads display either negative or positive correlations between lncRNA expression and the signal of epigenetic peaks (“–” and “+” peaks, respectively). To evaluate biological consistency with known data, we selected 30 lncRNAs and their corresponding histone peaks, for which “+” or “–” peaks of at least one histone mark are statistically significantly predominant (one-sided Fisher’s exact test, *p*-value < 0.001), confirmed by RNA-Chrom contacts extended by ±25 kb (see “Experimental Section,” subsection “One-Sided Fisher’s Exact Test,” *Fig. 4*). After filtering the results by a *p*-value < 0.001, we obtained the following “lncRNA–histone mark” pairs.

Twenty-one lncRNAs with “+” peaks of corresponding histone marks were better supported by RNA-Chrom contacts than “–” peaks (right-tailed Fisher’s exact test, *p*-value < 0.001).

Eleven lncRNAs with “–” peaks of corresponding histone marks were better supported by RNA-Chrom contacts than “+” peaks (left-tailed Fisher’s exact test,* p*-value < 0.001).

Previous studies have shown the potential involvement of many identified lncRNAs in epigenetic regulation via histone modifications. Let us consider cases where “+” peaks are statistically significantly better supported by RNA-Chrom contacts than “–” peaks. For example, MIR4435-2HG is involved in establishing the activator mark H3K27ac in the enhancer region of the RPTOR locus [29]. Our data suggest that MIR4435-2HG, in addition to H3K27ac, likely targets other epigenetic modifications, such as H3K27me3, H3K36me3, H3K4me1, H3K4me2, H3K4me3, and H3K79me2 (*Fig. 4A*). Similarly, based on the data for MIR31HG [30], SNHG1, PVT1 [31, 32, 33], and the mouse lncRNA lnc-Nr2f1 (presumed to have functional conservation with human NR2F1-AS1) [34], we identified consistent histone modifications: NR2F1-AS1 – H3K27ac, MIR31HG – H3K4me3, SNHG1 – H3K27me3, PVT1 – H3K27me3. Additionally, we uncovered functional associations of these lncRNAs with other epigenetic marks: NR2F1-AS1 – H3K4me2, MIR31HG – H3K79me2, SNHG1 – H3K4me3, PVT1 – H3K4me1, H3K4me3, H3K9me3 (*Fig. 4A*).

Numerous H3K27me3 and H3K4me3 “+” peaks, validated through a chromatin interaction analysis, were identified for several lncRNAs (ZFAS1, SNHG4, SNHG1, SNHG3, PVT1, MIR4435-HG, and CYTOR). These peaks exhibit significantly stronger support from RNA-chromatin contacts than “–” peaks (*Fig. 4A*), representing opposing chromatin states. By analogy with known lncRNAs that establish both marks depending on their association with different effector proteins (e.g., ncRNA SRA [35], ANRIL [36]), it can be hypothesized that these lncRNAs also exhibit more complex mechanisms of chromatin activity regulation.

Cases where “–” peaks are statistically significantly better supported by RNA-Chrom contacts than “”peaks can be explained by the corresponding lncRNAs regulating the removal of histone marks by recruiting demethylases and deacetylases to specific genomic loci (*Fig. 4B*). A lack of corroborating experimental data precludes a quality assessment of our predictions for these lncRNAs. We suggest that the “ncRNA–istone mark”pairs reported in this section (*Fig. 4*) are suitable candidates for further study.

**Usage examples**

The objective of integrating HiMoRNA and RNAChrom is to refine the functional relationship within the “lncRNA–pigenetic modification peak–ssociated gene” triads using data on the localization of the corresponding lncRNA in the genomic region near peaks of specific histone modifications. Below, we provide examples of user studies of several lncRNAs with known mechanisms of action.

**lncRNA MIR31HG**

The long non-coding RNA MIR31HG is a known regulator of the histone marks H3K4me1, H3K4me3, and H3K27ac. Previous studies have reported a reduction in the H3K4me1 and H3K27ac levels in the enhancer region of the *GLI2 *gene and H3K4me3 and H3K27ac in the promoter region of the *FABP4 *gene following MIR31HG knockdown [30, 37]. This observation can be validated using our integration of HiMoRNA and RNA-Chrom.

**Fig. 5:**
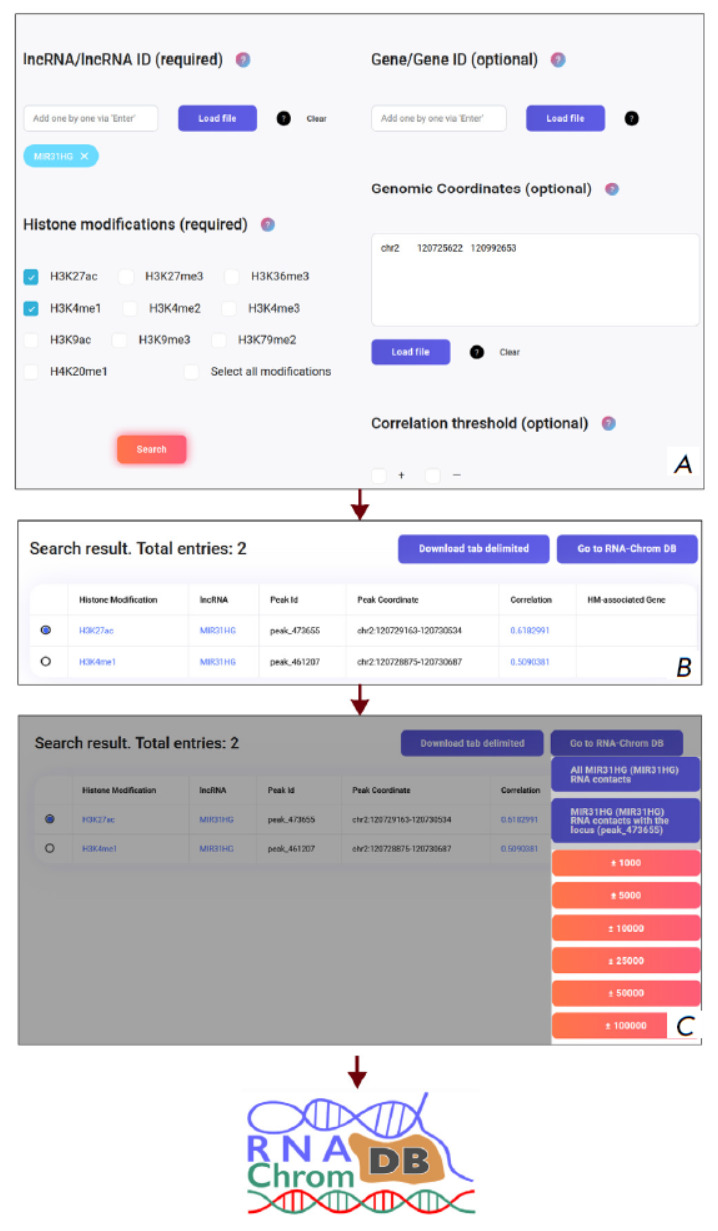
Use case of the integration of the HiMoRNA and RNA-Chrom databases using the example of lncRNA MIR31HG. (A) Creating a query in HiMoRNA for MIR31HG, histone modifications H3K4me1 and H3K27ac, and genes GLI2 and FABP4. (B) Table with search results. (C) Navigation to RNA-Chrom

**Fig. 6:**
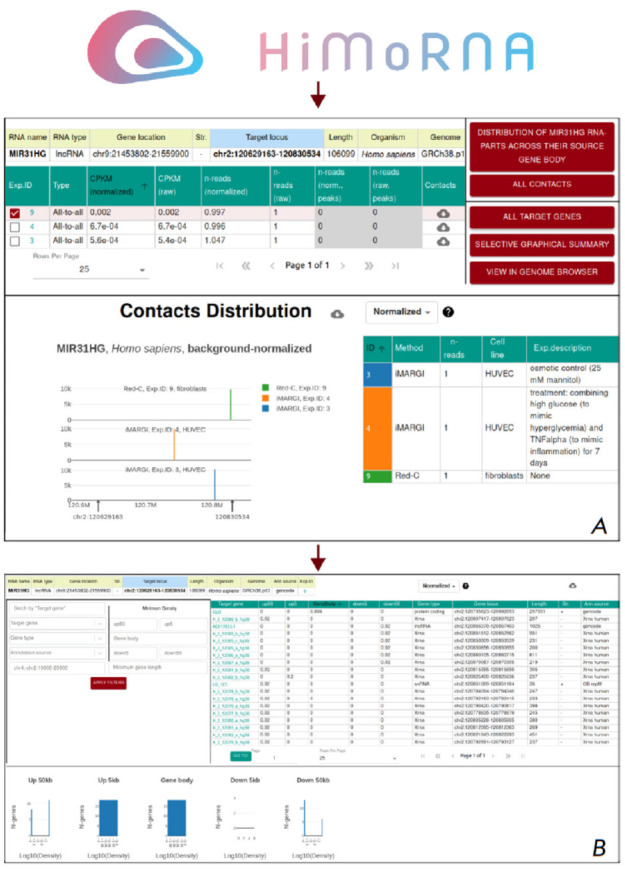
Use case of the integration of the HiMoRNA and RNA-Chrom databases using the example of lncRNA MIR31HG. (A) RNA-Chrom page showing MIR31HG contacts with chromatin in the region of the extended HiMoRNA peak. (B) Table listing all genes in the region of the extended peak, indicating whether they interact with MIR31HG or not (Experiment ID: 9)

To this end, we created a query in HiMoRNA: lncRNA MIR31HG, histone marks H3K4me1 and H3K27ac, with the coordinates of the two selected genes specified with an extended promoter region of 10 kb in the genomic coordinates field (*Fig. 5A*). As a result, the HiMoRNA web resource generated a table with H3K27ac and H3K4me1 peaks correlated with MIR31HG expression across various tissues (*Fig. 5B*). We then selected a triad with an H3K27ac peak and navigated to the RNA-Chrom page displaying experimentally detected MIR31HG chromatin contacts in the region of the selected peak (by clicking “Go to RNA-Chrom DB,” *Fig. 5C*). By selecting an RNA-chromatin experiment from the top table and clicking “All target genes” (*Fig. 6A*), we obtained a table that, in particular, reflected the interaction of MIR31HG with the *GLI2 *gene (*Fig. 6B*). A step-bystep analysis is presented in *Supplementary Table 3*.

To explore the potential for novel biological insights into the lncRNA function, we hypothesized that integrating the HiMoRNA and RNA-Chrom datasets would reveal that MIR31HG regulates additional components of the Sonic hedgehog signaling pathway (KEGG:04340) besides *GLI2*. For this purpose, we used the KEGG Pathway database [38] to identify relevant genes. Next, we formulated a new HiMoRNA query consisting of lncRNA MIR31HG, histone modifications H3K4me1 and H3K27ac, and the 56 genes associated with the Hedgehog signaling pathway (*Supplementary Table 4*). The outcome was a table of 162 triads, which can be validated with the RNA-Chrom resource. For example, in the locus of the H3K27ac_963553 peak (chr9:95446174-95452554), MIR31HG interacts with the *PTCH1 *gene, which encodes the “Sonic hedgehog” receptor. To determine if the gene set associated with the H3K27ac and H3K4me1 peaks, correlated with MIR31HG expression, exhibits significant enrichment of “Hedgehog signaling pathway” genes, a KEGG pathway enrichment analysis was conducted using the g:Profiler web resource [39]. The query included genes selected for MIR31HG and H3K27ac/H3K4me1, with all other genes associated with HiMoRNA peaks used as the background. The analysis revealed genes belonging to the “Hedgehog signaling pathway” to be enriched with H3K27ac peaks (*p*-value = 2.090 × 10-²) but not with H3K4me1 peaks. This observation suggests the involvement of MIR31HG in regulating the “Hedgehog signaling pathway” through the establishment of the H3K27ac histone modification in the corresponding genomic loci.

**lncRNA PVT1**

**Fig. 7:**
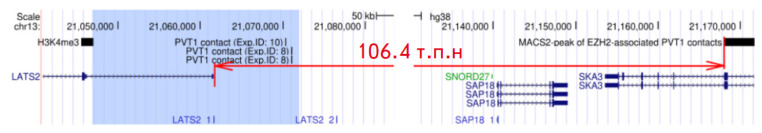
Representation in the UCSC Genome Browser of the region encompassing the LATS2 gene and its promoter vicinity, showing an H3K4me3 peak correlated with lncRNA PVT1 expression, lncRNA PVT1 contacts from two experiments (RNA-Chrom Exp. IDs: 8, 10), and an EZH2-mediated PVT1 contact peak. The blue region reflects the extension of the H3K4me3 peak coordinates by 25 kb, within which RNA-Chrom contacts were selected

The long non-coding RNA PVT1 is known to inhibit the expression of the *LATS2 *gene in non-small cell lung cancer cells by recruiting EZH2 (a subunit of the PRC2 complex) to the corresponding promoter [40]. We performed a search for triads in HiMoRNA: lncRNA PVT1, all histone modifications, and the *LATS2 *gene. Our findings revealed solely H3K4me3 activation peaks, exhibiting a negative correlation with PVT1 expression levels. This observation is consistent with previously published findings [39], wherein PVT1’s recruitment of EZH2 contributes to the establishment of the repressive H3K27me3 mark. In RNA-Chrom, we observed contacts around one of the H3K4me3 peaks (peak_169403, chr13:21045571-21046978) in two experiments (K562 and MDA-MB-231 cell lines). Visualization of PVT1 contacts in the Genome Browser [41] confirms the presence of this peak in the promoter region of the* LATS2 *gene (*Figure 7*, step-by-step analysis presented in *Supplementary Table 5*). Additional confirmation of *LATS2 *regulation by lncRNA PVT1 was obtained using Red-ChIP data (see “Experimental Section,” subsection “Red-ChIP Data”). A peak of EZH2- mediated PVT1 contacts (chr13:21168000-21224000, q-value = 0.09) was identified 106.4 kb from the 5’- end of the *LATS2 *gene (*Figure 7*).

The absence of “lncRNA PVT1–H3K27me3 peak–* LATS2 *gene” triads with a positive correlation in HiMoRNA is likely due to overly stringent filtering of H3K27me3 peaks during the database creation. The preceding examples from the “Use Cases” section illustrate the successful application of this integration in generating testable hypotheses regarding lncRNA involvement in the epigenetic regulation of specific genes.

## DISCUSSION

**Prospects for Use and Limitations of the Approach**

The HiMoRNA database comprises genomic loci exhibiting a significant correlation between histone modification signals and lncRNA expression across diverse cell types and tissues. Currently, it contains over 5 million correlations for 10 types of histone modifications and 4,145 lncRNAs. We hypothesize that some of these correlations may represent false positives or indirect regulatory relationships, thus requiring further validation using external data. The RNA-Chrom database houses genome-wide RNA-chromatin interaction data. These data unfortunately lack sufficient representation of contacts from lowly expressed ncRNAs and are heavily biased towards contacts from nascent transcripts. Furthermore, these data are insufficient to formulate hypotheses regarding the functional roles of these interactions. With these limitations in mind, integrating the data from HiMoRNA and RNA-Chrom is a reasonable approach to characterize the impact of lncRNAs on epigenetic modifications and gene expression.

Despite the advantages of integration, some inconsistencies remain. No H3K27ac or H3K4me1 peaks were found in the *FABP4 *gene locus in the HiMoRNA database. This observation contradicts experimental data and the RNA-Chrom database. There are other negative examples in both HiMoRNA and RNAChrom for well-known lncRNAs involved in epigenetic regulation and chromatin structure maintenance. For instance, no H3K27me3 peaks correlating with MEG3 were observed in HiMoRNA, despite evidence suggesting that MEG3 regulates the PRC2 complex and contributes to maintaining H3K27me3, particularly in the promoter regions of the *SMAD2*, *TGFB2*, and *TGFBR1 *genes [11]. The absence of such peaks is likely attributable to the cell-specific expression of most lncRNAs, with the mentioned mechanism observed in a cell type not represented in HiMoRNA. Even when ChIP-seq data are available, the standard peak-calling procedure may be too stringent, potentially filtering out biologically significant interactions.

We observed instances where HiMoRNA predictions aligned with published experimental data, but the corresponding RNA-chromatin contacts were absent from the relevant genomic locus in the RNA-Chrom dataset. For example, for lncRNA MAPKAPK5-AS1, most correlated peaks from HiMoRNA are not supported by the expression of this RNA, resulting in a low number of observed chromatin contacts in “all-to-all” experiments. We posit that these instances may stem from variations in cell types across the two databases as a result of insufficient data.

Due to insufficient experimental data, neither database contains exhaustive information. Therefore, some documented biological examples might have been overlooked in the integration process. Nevertheless, their integration offers complementary strengths, such as mitigating various systematic errors that are due to the multi-omics nature of the combined data and expanding the generation of interpretable hypotheses about the mechanisms of epigenetic regulation of gene expression by long non-coding RNAs.

## FURTHER DEVELOPMENT

The implemented integration could be significantly improved through the incorporation of supplementary genome-wide data and annotations. The dataset may contain information regarding the three-dimensional chromatin structure, gene expression and co-expression patterns (including long non-coding RNA expression, the target genes of the triad, and the genes associated with histone modification), in addition to the localization of DNA-binding and chromatin- modifying proteins. Given the current scarcity of such experimental data, it is worth considering the use of bioinformatics predictions. One potential direction could involve incorporating results from predictions of the type of lncRNA-chromatin interactions (for a comparison of programs determining the mechanisms of lncRNA interactions with other molecules; see, for example, [42]). The combination of predicted lncRNA–target interactions and multi-omics experimental data has facilitated effective hypothesis generation concerning the roles of specific lncRNAs [43, 44, 45]. Another important direction would be to include data on gene expression changes following artificial alterations in the concentration of specific lncRNAs in cells [8], as well as experimentally validated information on the involvement of specific lncRNAs in regulating particular histone modifications [46]. This would provide an additional layer of validation for the results of the HiMoRNA and RNA-Chrom integration. Furthermore, from a practical perspective, it would be useful to enhance the integration with an assessment of the statistical significance of the co-localization of HiMoRNA peaks and RNA-Chrom contacts for a specific lncRNA using specialized software tools such as Genometricorr [47], StereoGene [48], and RegioneR [49].

The field of lncRNA research is rapidly evolving. We will maintain support for the integration of HiMoRNA and RNA-Chrom as both databases expand their taxonomic scope and incorporate updated data. Upon the emergence of new, experimentally validated data, we intend to construct multiple predictive models for “lncRNA–histone epigenetic modifications– associated gene” interactions. We are confident that the continued collaborative expansion of the HiMoRNA and RNA-Chrom web resources will contribute to a deeper understanding of the functional role of lncRNAs in the epigenetic regulation of genes.

## Abbreviations

ncRNA: non-coding RNA
lncRNA: long non-coding RNA
